# Supplementary material for: Brain cell type specific proteomics approach to discover pathological mechanisms in the childhood CNS disorder mucolipidosis type IV
Source: Front Mol Neurosci. 2023 Aug 7;16:1215425. doi: 10.3389/fnmol.2023.1215425 (PMC10440433; doi:10.3389/fnmol.2023.1215425)
Supplement: Supplementary file 6 [file Data_Sheet_1.docx]

Supplementary Material

Brain cell type specific proteomics approach to discover pathological mechanisms in the childhood CNS disorder mucolipidosis type IV.

Madison Sangster ^1#^, Sanjid Shahriar^2#^, Zachary Niziolek^3^, Maria Carla Carisi ^1^, Michael Lewandowski^2^, Bogdan Budnik^2*^and Yulia Grishchuk^1*^

*** Correspondence:** [bogdan.budnik@wyss.harvard.edu](mailto:bogdan.budnik@wyss.harvard.edu) **and** [ygrishchuk@mgh.harvard.edu](mailto:ygrishchuk@mgh.harvard.edu)

# Supplementary Tables

**Supplementary Table 1. FACS-sorted brain cells count from *Mcoln1^-/-^* and control mice.**

| Mouse | Genotype | Cell counts, x10^3^ cells | | | | |
| --- | --- | --- | --- | --- | --- | --- |
|  |  | Neurons | NSC | Oligodendrocytes | Astrocytes | Microglia |
| 1 | Mcoln1 +/- | 822 | 5.2 | 2300 | 268 | 13 |
| 2 | Mcoln1 +/- | 600 | 14 | 2400 | 167 | 28 |
| 3 | Mcoln1 +/- | 634 | 42 | 908 | 311 | 161 |
| 4* | Mcoln1 +/- | 45 | 5 | 287 | 75.7 | 8.5 |
| 5 | Mcoln1 +/- | 2000 | n/a | 2750 | 236 | 187 |
| 6 | Mcoln1 -/- | 749 | 162 | 680 | 137 | 166 |
| 7 | Mcoln1 -/- | 1400 | 87 | 678 | 177 | 119 |
| 8 | Mcoln1 -/- | 360 | 94 | 560 | 100 | 97 |
| 9 | Mcoln1 -/- | 287 | 240 | 529 | 121 | 220 |
| 10 | Mcoln1 -/- | 1.64 | 41 | 300 | 376 | 153 |
|  | Mean (SEM) | **761.5 (+/- 202.5)** | **85.65 (+/- 28.4)** | **915 (**+/- **276)** | **210.3 (**+/- **31.1)** | **127 (+/- 23.4)** |

*sample removed from data analysis

**Supplementary Table 2. Protein abundances in whole cortical homogenates and isolated brain cells in *Mcoln1^-/-^* mice.**

**Supplementary Table 3. Typical protein contaminants excluded from the proteomics data sets.**

**Supplementary Table 4. Overlapping and non-overlapping proteins in all sample types.**

**Supplementary Table 5. Pathway analysis outcomes in all sample types.**

**Supplementary Table 6. Overlapping and non-overlapping enriched pathways in all sample types.**

## Supplementary Figures

**Supplementary Figure 1.** Principal component analysis (PCA) showing separation of *Mcoln1^-/-^* samples from controls.


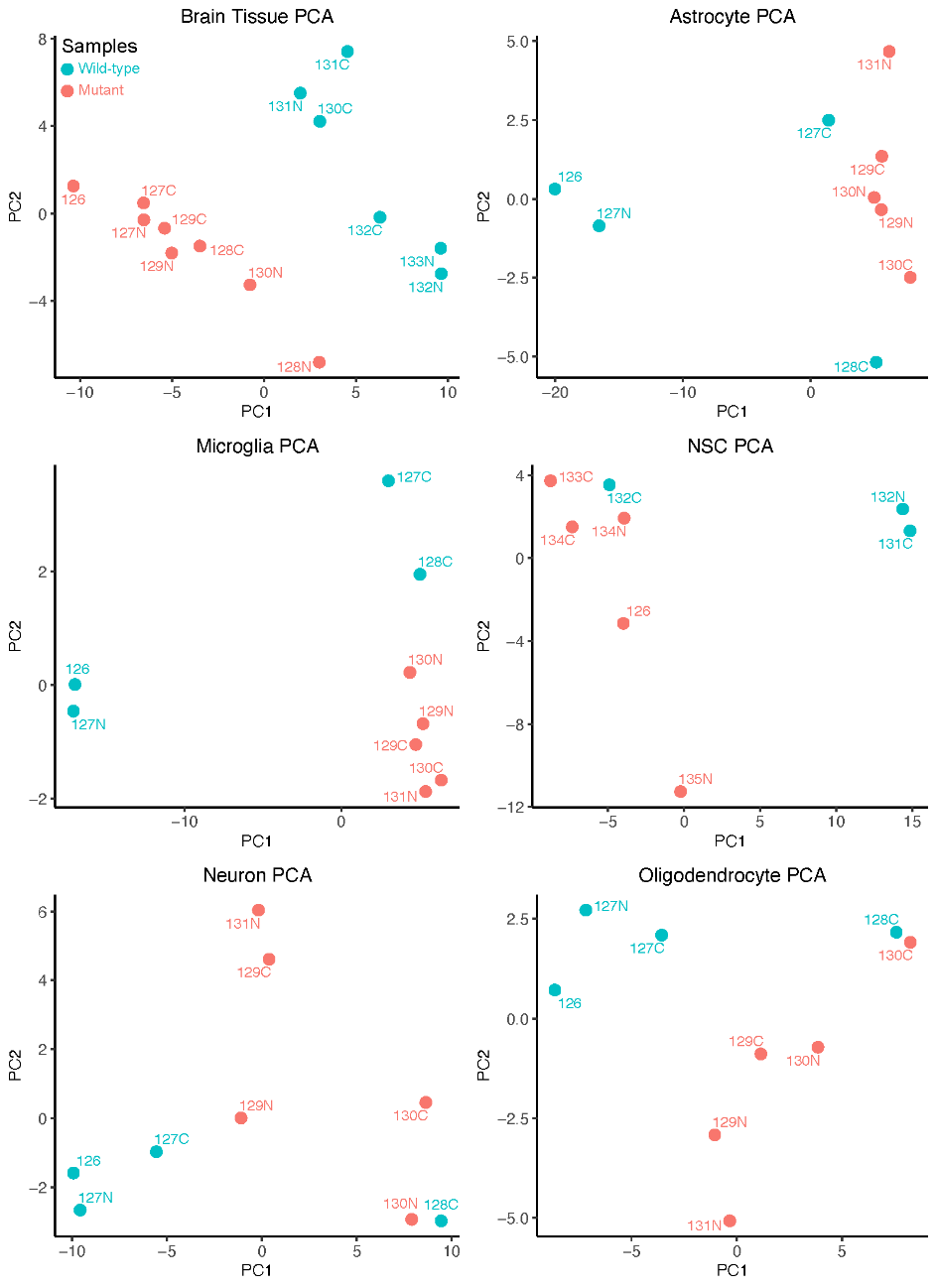


# Data Availability

The data can be found at the following link: <https://massive.ucsd.edu/ProteoSAFe/dataset.jsp?accession=MSV000091824>

MassIVE MSV000091824, PXD041888, doi: 10.25345/C5QV3CD6M
